# Association between the risk of relative energy deficiency in sport and cholesterol levels in Finnish endurance athletes

**DOI:** 10.1136/bmjsem-2025-002644

**Published:** 2025-09-01

**Authors:** Julia I K Silvennoinen, Pyry N Sipilä, Maarit Valtonen, Katja Mjøsund, Ville Kinnula, Leon Hirvelä, Laura Mierlahti, Johanna K Ihalainen

**Affiliations:** 1 Department of Public Health, University of Helsinki Faculty of Medicine, Helsinki, Finland; 2 University of Jyväskylä Faculty of Sports and Health Sciences, Jyväskylä, Finland; 3 Finnish Institute of High Performance Sport KIHU, Jyväskylä, Finland; 4 Paavo Nurmi Centre and Unit for Health and Physical Activity, University of Turku, Turku, Finland; 5 National Olympic Training Center, Helsinki, Finland; 6 Biostatistics Unit, Helsinki University Central Hospital, Helsinki, Finland; 7 Biostatistics Unit, University of Helsinki, Helsinki, Finland; 8 Biology of Physical Activity, University of Jyväskylä, Jyväskylä, Finland

**Keywords:** Cholesterol, Eating disorders, Sports medicine, Sports and nutrition, Female athlete triad

## Abstract

**Background:**

Relative energy deficiency in sport (REDs) is a condition caused by chronic and/or severe low energy availability. Endurance athletes are at risk of REDs, which are characterised by negative effects on health and performance. Disturbed cholesterol metabolism is a suggested indicator of REDs and could affect the future cardiovascular health of athletes. We investigated the association between the REDs risk and cholesterol levels in endurance athletes.

**Methods:**

Finnish endurance athletes (n=87; female 44, male 43) were recruited as part of the Athletic Performance and Nutrition study. The participants were examined at the beginning (T1) and end (T2) of the training season. The REDs risk was assessed using the REDs Clinical Assessment Tool version 2.

**Results:**

At T1, 37 (77%) female and 31 (72%) male athletes were at medium-to-high REDs risk. The REDs risk was not associated with cholesterol levels in a cross-sectional analysis, and no overall change in the REDs risk or the cholesterol levels was observed between T1 and T2. In female athletes, an increase in REDs risk status from low to medium-to-high between T1 and T2 was associated with a decrease in low-density lipoprotein (LDL) cholesterol (beta adjusted for age −0.62, 95% CI −0.94 to −0.30) and total cholesterol (beta adjusted for age −0.85, −1.42 to −0.28)).

**Conclusions:**

REDs risk was not associated with cholesterol levels at T1 or T2. However, in female athletes, an increase in the REDs risk across time points was associated with a decrease in LDL and total cholesterol levels.

**WHAT IS ALREADY KNOWN ON THIS TOPIC**

- Disturbed cholesterol metabolism is one of the potential clinical manifestations of relative energy deficiency in sport (REDs) and could affect the future cardiovascular health of athletes.

**WHAT THIS STUDY ADDS**

- Cholesterol levels were not associated with REDs risk assessed with REDs Clinical Assessment Tool version 2, but an increase in the REDs risk across time points was associated with a decrease in low-density lipoprotein (LDL) and total cholesterol levels in female athletes.
- The prevalence of elevated LDL cholesterol was similar in endurance athletes as in the general population.

**HOW THIS STUDY MIGHT AFFECT RESEARCH, PRACTICE OR POLICY**

- Our study suggests that the inclusion of elevated LDL and total cholesterol in the REDs screening tool may require further evaluation.
- Athletes should not automatically be assumed to be at low cardiovascular risk, as a substantial proportion of athletes had clinically elevated LDL and total cholesterol levels.

## Background

 Problematic low energy availability (LEA) is a condition where an athlete’s energy consumption chronically and/or severely fails to meet energy demands of both exercise and basic physiological functions.^1^ Relative energy deficiency in sport (REDs) is a model to illustrate the wide-ranging negative effects problematic LEA can have on various physiological systems, health and performance. REDs comprises the effects of LEA on hormonal regulation, bone health, immune function, reproductive system, cardiovascular health and cholesterol metabolism in both female and male athletes.^1^

LEA can suppress the hypothalamic–pituitary–gonadal axis, leading to hormonal disturbances such as low oestrogen in females^2^ and low testosterone in males.^3^ These two reproductive hormones have been associated with beneficial cardiovascular and lipid outcomes, although current evidence for testosterone is mixed. Oestrogen protects the cardiovascular system by preventing the formation of atherosclerotic lesions,^4^ in part by improving hepatic reverse cholesterol transport.^5^ Testosterone is also thought to protect against atherosclerotic heart disease even if males have a higher cardiovascular risk than premenopausal females.^5^ Androgen receptor signalling appears to influence insulin sensitivity, glucose metabolism, obesity and muscle mass, potentially contributing to better cardiovascular health.^5 6^

Although the REDs model suggests a potential link between problematic LEA and disruptions in cholesterol metabolism, previous findings on the association are conflicting. Some studies have reported elevated low-density lipoprotein cholesterol (LDL-C) or total cholesterol (TC) in athletes at risk of LEA,^27^,^13^ while others have not observed this.^714^,^18^ Disordered eating behaviour in female athletes and eating disorders in the general population have been linked to higher LDL-C and TC levels.^7 19^

Recognising the need for further research on the interplay between LEA and cardiovascular health, and to extend our previous work,^7^ we investigated the association between the REDs risk, assessed using the REDs Clinical Assessment Tool version 2 (REDs CAT2), currently recommended by the International Olympic Committee (IOC),^1^ and cholesterol levels in female and male endurance athletes. We focused on seasonal changes in cholesterol levels and the REDs risk, as well as their associations. Our hypothesis was that higher REDs risk would be associated with higher LDL-C and TC levels throughout the athletic year.

## Methods

### Study design and recruitment of participants

We used data from the Athletic Performance and Nutrition (NoREDS) study conducted at the University of Jyväskylä, Finland. NoREDS was a 3-year follow-up study that analysed the body composition, and energy and nutrient intake of athletes at the beginning (T1) and end (T2) of their training season. At T1, the athletes had recently finished their off-season and were at the beginning of their general training phase. T2 took place at the end of the specific training phase before the start of the competition season. A total of 391 Finnish national (tier 3) to world class (tier 5) level female and male athletes,^20^ aged 16–35 years and without acute injuries or previous severe medical conditions at baseline, were included in the study and were followed up from 2021 to 2024. The use of hormonal contraception was not an exclusion criterion, but a sensitivity analysis was conducted to account for the potential confounding effect. Female athletes using hormonal contraception were excluded from the sensitivity analysis regarding the menstrual status.

For the present study, we included adult (≥18 years) female and male athletes from endurance sports (long distance running, cross country skiing, Nordic combined, biathlon, triathlon, orienteering and race walking). For the first part of the study, we included the first measurements of all athletes from T1 and T2 for two separate cross-sectional analyses. For the second part of this study, we included participants who had completed two consecutive measurements within one athletic season (T1+T2). Participants with missing data on key variables (cholesterol values, bone mineral density (BMD), triiodothyronine (T3), testosterone, eating disorder symptoms and body mass index) were excluded (n=7). Thus, the final sample comprised 87 (T1), 71 (T2) and 58 (T1+T2) athletes (online supplemental figure S1). Athletes in the longitudinal analysis represented the same sports as the athletes in the cross-sectional analysis.

### Measures

#### Blood measurements (cholesterol and hormone levels)

Fasting venous blood samples were drawn from the antecubital vein in a supine position between 7:00 and 10:00 AM. For serum separation, whole blood was left to clot for 30 min at room temperature and centrifuged at 2200×g before aliquoting and storing the sera at −80°C until analysis. Serum triiodothyronine (T3) and testosterone concentrations were determined using chemiluminescent enzyme immunoassay IMMULITE 2000 XPi (Siemens Healthcare Diagnostics, UK). Serum high-density lipoprotein (HDL-C), LDL-C and TC were measured using a KONELAB 20 XTi or Indico analyser (Thermo Fischer Scientific, Finland) according to the manufacturer’s instructions.

#### Eating disorder symptoms

The Eating Disorder Examination Questionnaire Short (EDE-QS), a shorter version of the original Eating Disorder Examination Questionnaire (EDE-Q), was used to assess eating disorder symptoms.^21^ The EDE-QS assesses the severity, frequency and extent of eating disorder symptoms during the past 7 days using 12 items and a 4-point scale.^22^ This yields total scores ranging from 0 to 36, with higher scores indicating more unhealthy attitudes towards eating and body image. The EDE-QS has demonstrated high internal consistency (Cronbach’s alpha=0.91) and convergent validity with the EDE-Q among those with (r=0.91) and without (r=0.82) an eating disorder.^23^ We used a cut-off of ≥15, which has been recommended for screening for a clinical eating disorder.^22^

#### Risk of REDs

We used the REDs CAT 2^24^ to identify athletes at risk of REDs. Athletes were initially categorised by the REDs CAT 2 into a traffic light system from green to yellow, orange or red depending on the severity of the risk as presented in tables1  2. As there was a low number of individuals categorised as orange or red, we analysed the athletes in two groups: low REDs risk, which included the athletes categorised as green, and medium-to-high REDs risk, which included athletes categorised as yellow, orange or red.

**Table 1:** Primary and secondary indicators of REDs used in the present study

Severe primary indicators (count as two primary indicators)	Amenorrhoea for females (absence of menstrual cycle for >3 months). Clinically low total testosterone (<10 nmol/L) for males.
Primary indicators	Subclinically low free or total testosterone (10–17 nmol/L) for males. Subclinically or clinically low total T3 (≤4.25 pmol/L). History of ≥1 high-risk (femoral neck, total hip, sacrum, pelvis) or ≥2 low-risk (all other locations) bone stress injuries (BSI) within the previous 2 years. Bone mineral density Z-score <−1 at the lumbar spine (L1−L4) or femoral neck. Eating Disorder Examination Questionnaire Short score ≥15.
Secondary indicators	Oligomenorrhoea for females (<8 menstrual cycles in the past 12 months). History of 1 low-risk BSI within the previous 2 years.

Indicators and reference levels are the same for female and male participants unless otherwise indicated.

REDs, relative energy deficiency in sport.

**Table 2:** Risk assessment of REDs used in the present study

Red/severe risk	3 primary and ≥2 secondary, or ≥4 primary indicators.
Orange/high risk	1 primary and ≥3 secondary, or 2 primary and ≥2 secondary, or 3 primary and ≤1 secondary indicators.
Yellow/mild risk	0 primary and ≥2 secondary, or 1 primary and ≤2 secondary, or 2 primary and ≤1 secondary indicators.
Green/low risk	0 primary ≤1 secondary indicators.

The data were analysed as low risk (green) vs medium-to-high risk (yellow, orange or red) because very few athletes were categorised as orange or red.

REDs, relative energy deficiency in sport.

Due to the enrolment of our study prior to the publication of the REDs CAT 2, the indicators of the REDs risk in this study had some differences with the published tool.^24^ Information about the duration of amenorrhoea was not collected and therefore, all forms of amenorrhoea were categorised as a severe primary indicator, which in the published tool included any primary amenorrhoea and secondary amenorrhoea lasting for at least 12 months. We also lacked the data to assess anxiety and depression. We had a more concise version of EDE-Q, the EDE-QS, for the measure of disordered eating behaviour. As all our participants were ≥18 years old, we excluded the criteria for adolescents and children. Finally, we excluded cholesterol levels from the risk assessment as our aim was to investigate the association between REDs risk and cholesterol levels.

#### Maximal oxygen uptake

A treadmill test was performed using a standard incremental protocol as previously described.^25^ Maximal oxygen uptake (VO_2_ max) was defined as the highest average 60 s VO_2_ value measured breath-by-breath using a portable gas analyser.

#### Anthropometrics and BMD

Anthropometric measurements were carried out between 7:00 and 10:00 AM after a fasting period of 10 hours; only small amounts of water (maximum 2 dL) were allowed to be consumed in the morning. Participants were advised to refrain from strenuous physical activity the preceding day and the morning of the measurement and to go to the toilet before the measurement. Dual Energy X-ray Absorptiometry (DXA, LUNAR Prodigy, GE Healthcare, Madison, Wisconsin, USA) was used to assess body composition and bone properties. BMD was evaluated using Z-scores. Fat mass to lean mass ratio (FLMR) was calculated by dividing the total fat mass by the total lean mass. The device manufacturer’s standard procedures were followed, and the device was calibrated with a phantom each morning prior to the measurements for quality assurance. Participants were scanned in supine position in the centre of the table using the default-scan mode automatically selected by the Prodigy software (Lunar Prodigy Advance Encore V.14.10.022). Athletes underwent whole-body DXA scans at both T1 and T2. However, due to ethical considerations, site-specific BMD assessments of the lumbar spine and proximal femur were performed only once per athletic year. These values were extrapolated to represent both time points within the same annual cycle.

#### Season of the year

To account for potential variance in cholesterol levels due to the season of the year, we classified the time points of measurements into four categories using the ‘light season definition’ (winter: 6 November to 4 February; spring: 5 February to 6 May; summer: 7 May to 5 August; fall: 6 August to 5 November).^26 27^

### Statistical analysis

Data analysis was conducted in R (V.2024. R 4.4.1 GUI 1.80). All analyses were conducted separately for females and males. The normality of the parameters was assessed visually. Normally distributed variables were analysed using parametric tests and non-normally distributed variables using non-parametric tests. Results are reported as means±SD (normally distributed data), or median and IQR (non-normally distributed data). All CIs are reported at the 95% confidence level. The significance level (α) was set to 0.05. To assess the risk of type II error, we estimated the minimum detectable effect (MDE) for each analysis with 80% power at α=0.05, based on the actual sample sizes in each analysis.

The significance of the changes from T1 to T2 was analysed either with Student’s paired t-test (normally distributed data), Wilcoxon signed rank test (non-normally distributed data) or McNemar’s test (for categorical data). MDE was 0.56 SD for females and 0.52 SD for males in the continuous variables. For the REDs risk, we were powered to detect a change of 45% for female athletes and 32% for male athletes between T1 and T2. To further investigate changes over time points of the training season in the study parameters, we fit a separate linear mixed model for each parameter with fixed effects for the time point, as well as age for adjustment.^28^ Random intercepts for each participant were also specified to account for individual variability. The model was estimated using restricted maximum likelihood.^29^ Cross-sectional relationships between REDs risk and each of HDL-C, LDL-C and TC were assessed using separate linear regressions adjusted for age. HDL-C, LDL-C and TC were the outcome variables and REDs risk was the explanatory variable in our analysis. MDE of unstandardised beta was 0.40–1.08 mmol/L for females and 0.39–1.08 mmol/L for males (online supplemental table S9).

To analyse the association of changes (Δ) seen from T1 to T2, we fit a linear regression model between the change observed in HDL-C, LDL-C or TC and REDs risk within the athletes who had participated in both measurements in the training season. The change in cholesterol levels was calculated by subtracting the value measured in the beginning of the training season from the value measured at the end of the training season (Δ=T2–T1). The change in REDs risk was defined as a shift between risk categories (low ↔ medium-to-high) calculated individually (Δ=T2–T1), with all directional changes included in the analysis. These models were also adjusted for age. Sensitivity analysis was further adjusted for change in body fat percentage, FLMR and season of the year. MDE of unstandardised beta was 0.35–0.88 mmol/L for females and 0.28–0.78 mmol/L for males (online supplemental table S10). The assumptions of all models were examined by visual inspection of residuals. The statistical analysis was reviewed to be consistent with the CHAMP statement.^30^ We used the Strengthening the Reporting of Observational Studies in Epidemiology cross-sectional checklist when writing our report.^31^

### Equity, diversity and inclusion statement

The authors are all based in Nordic countries and comprise both women and men and include both junior and senior researchers. The authors have backgrounds as medical doctors, exercise physiologists, biostatisticians or sport dietitians. Our study population included athletes of different ages and both sexes.

## Results

### Participant characteristics

Our analytical sample comprised 44 female and 43 male athletes at the beginning (T1), and 36 female and 35 male athletes at the end (T2) of the training season. In total, 27 female and 31 male athletes had completed measurements in both time points (T1+T2) and were included in the longitudinal analysis with a mean 4.4 months (SD 1.3) time difference between T1 and T2. VO_2_ max increased among females (p=0.002) and body fat percentage (p=0.013) and FLMR (p=0.019) increased among males from T1 to T2 (online supplemental table S1). Further characteristics of the participants are described in table 3, online supplemental table S1.

**Table 3:** Characteristics of study participants

	Female athletes				Male athletes			
	n	T1	n	T2	n	T1	n	T2
Age	44	23.9±3.9	36	24.4±4.2	43	24.7±3.5	35	25.5±3.6
BMI	44	20.6±1.4	36	20.7±1.6	43	22.3±1.8	35	22.3±1.5
Fat %	44	16.4 (12.7–19.3)	36	15.7 (12.8–19.5)	43	7.4 (5.8–10.0)	35	8.2 (6.9–10.7)
FLMR	43	0.19±0.06	35	0.20±0.08	43	0.087±0.03	43	0.10±0.04
Medium-to-high REDs risk	44	34 (77%)	36	26 (72%)	43	31 (72%)	35	30 (86%)
Amenorrhoea/ oligomenorrhoea	33	12 (36%)	27	<5 (<19%)	–	–	–	–
HDL-C	44	1.8 (1.6–2.1)	36	1.8 (1.6–2.1)	43	1.5 (1.3–1.9)	35	1.5 (1.3–1.8)
LDL-C	44	2.4±0.7	36	2.6±0.9	43	2.3±0.6	35	2.5±0.6
TC	44	4.4±0.9	36	4.7±1.1	43	4.1±0.8	35	4.3±0.8
VO_2_ max	35	59.8±5.0	25	61.5±5.3	40	70.7±5.8	31	70.9±6.0

Data are mean ±SD, median (IQR) or n (%).

BMI, body mass index (kg/m2); FLMR, fat mass to lean mass ratio; HDL-C, high-density lipoprotein cholesterol; LDL-C, low-density lipoprotein cholesterol; REDs, relative energy deficiency in sport; T1, beginning of the training season; T2, end of the training season; TC, total cholesterol ; VO_2_max, maximal oxygen uptake .

### REDs risk

**Figure 1:**
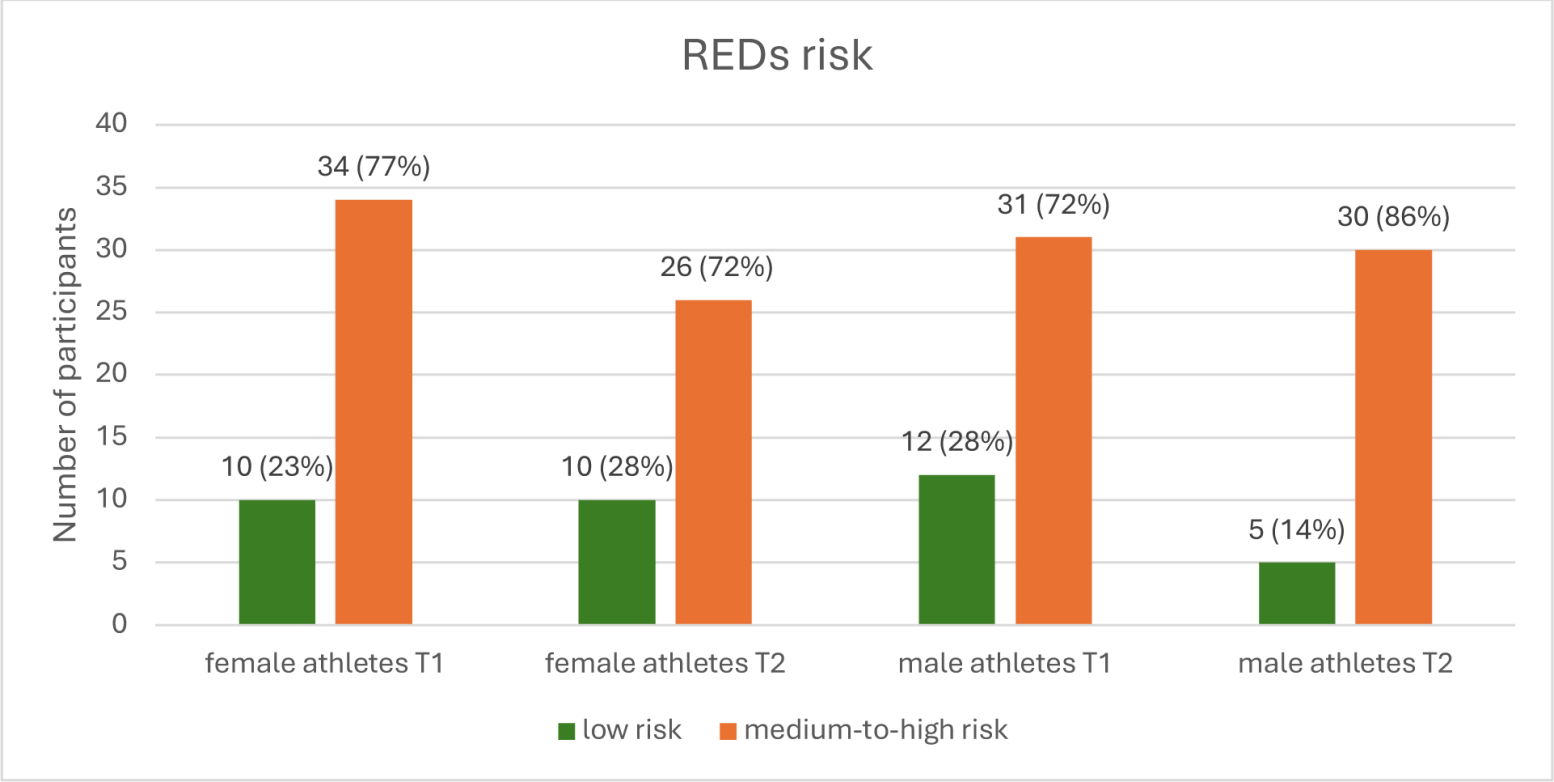
REDs risk in female and male athletes at the beginning (**T1**) and end (**T2**) of the training season. REDs, relative energy deficiency in sport.

Both at the beginning (T1) and at the end (T2) of the training season, over 70% of both female and male athletes were at medium-to-high REDs risk (figure 1). The prevalences in the longitudinal analysis are presented in online supplemental figure S2. No significant change in REDs risks was observed between T1 and T2 (p=0.75 for females and p=0.18 for males, online supplemental table S1.

### Clinically elevated cholesterol levels

At T1, 7 (16%) of the 44 female athletes and 5 (12%) of the 43 male athletes had a clinically elevated LDL-C (≥3 mmol/L). At T2, 11 (31%) of the 36 female athletes and 7 (20%) of the 35 male athletes had a clinically elevated LDL-C. At T1, 13 (30%) of the 44 female athletes and 5 (12%) of the 43 male athletes had a clinically elevated TC (≥5 mmol/L). At T2, 13 (36%) of the 36 female athletes and 7 (20%) of the male athletes had TC≥5 mmol/L.

### REDs risk and cholesterol levels

We observed no association between REDs risk and HDL-C, LDL-C, or TC at either T1 or T2 (table 4). As for individual REDs indicators, HDL-C was negatively associated with T3 in males in both T1 and T2 (online supplemental table S2). No association was found between LDL-C or TC and individual REDs indicators (online supplemental tables S3 and S4).

**Table 4:** Association of HDL-C, LDL-C and TC with REDs risk in female and male athletes in a cross-sectional analysis at the beginning (T1) and end (T2) of the training season

	n	beta adjusted for age	95% CI	P value
Females T1				
HDL-C	44	0.19	−0.097 to 0.47	0.19
LDL-C	44	−0.027	−0.53 to 0.48	0.92
TC	44	0.045	−0.65 to 0.096	0.90
Females T2				
HDL-C	36	0.074	−0.28 to 0.43	0.68
LDL-C	36	−0.28	−0.90 to 0.35	0.38
TC	36	−0.32	−1.13 to 0.50	0.43
Males T1				
HDL-C	43	0.064	−0.22 to 0.35	0.66
LDL-C	43	−0.049	−0.48 to 0.38	0.82
TC	43	−0.15	−0.70 to 0.40	0.58
Males T2				
HDL-C	35	0.091	−0.21 to 0.39	0.54
LDL-C	35	0.14	−0.41 to 0.69	0.60
TC	35	0.20	−0.53 to 0.94	0.57

HDL-C, high-density lipoprotein cholesterol (mmol/l); LDL-C, low-density lipoprotein cholesterol (mmol/l); REDs, relative energy deficiency in sport; TC, total cholesterol (mmol/l).

### Change in cholesterol levels between T1 and T2

No significant change was observed in HDL-C, LDL-C, or TC between T1 and T2 (online supplemental tables S1 and S5). The associations between changes (Δ) in REDs risk and cholesterol levels between T1 and T2 are shown in table 5. In female athletes, we observed a negative association between the Δ REDs risk with the Δ LDL-C (adjusted beta –0.62, 95% CI –0.94 to –0.30) and the Δ TC (–0.85, 95% CI –1.42 to –0.28).

**Table 5:** Association of the change in REDs risk from the beginning (T1) to the end (T2) of the training season with the change in cholesterol values

Δ REDs risk	n	beta unadjusted	95% CI	P value	beta adjusted for age	95% CI	P value
Females							
Δ HDL-C	27	−0.13	−0.38 to 0.12	0.30	−0.15	−0.029 to 0.061	0.25
Δ LDL-C	27	−0.60	−0.91 to −0.28	**0.00056**	−0.62	−0.94 to −0.30	**0.00049**
Δ TC	27	−0.79	−1.35 to −0.22	**0.0082**	−0.85	−1.42 to −0.28	**0.0050**
Males							
Δ HDL-C	31	−0.10	−0.31 to 0.10	0.31	−0.11	−0.34 to 0.12	0.35
Δ LDL-C	31	0.22	−0.19 to 0.62	0.28	0.21	−0.25 to 0.66	0.36
Δ TC	31	0.031	−0.55 to 0.61	0.92	0.063	−0.59 to 0.11	0.85

Bolded p values are below the set statistical significance level of 0.05.

HDL-C, high-density lipoprotein cholesterol; LDL-C, low-density lipoprotein cholesterol; REDs, relative energy deficiency in sport; TC, total cholesterol.

### Sensitivity analysis

11 female athletes (25%) reported the use of hormonal contraception in T1 and 8 (22%) in T2. 19 female athletes not using hormonal contraception were included in the longitudinal sensitivity analysis (T1+T2). The results were similar with the main analysis. We observed no association between the REDs risk and HDL-C, LDL-C or TC at either T1 or T2 (online supplemental table S6), nor between the menstrual status and HDL-C, LDL-C or TC at either T1 or T2 (online supplemental tables S2–S4). Δ REDs risk had a negative association with Δ LDL-C and Δ TC (online supplemental table S7).

We also conducted sensitivity analyses to account for the potential effects of body composition or season of the year (winter, spring, fall, summer). The main findings reported in table 5 remained similar after additional adjustment for changes in body fat percentage or FLMR (online supplemental table S8). Also, the associations of change in REDs risk with change in LDL-C and TC were significant among females both in the main models (table 5) and after further adjustment for the season of the year (ΔLDL-C beta −0.55, 95% CI −0.88 to −0.22), p=0.0020; ΔTC beta −0.79, 95% CI −1.39 to −0.19, p=0.012).

## Discussion

We investigated the association between REDs risk and cholesterol levels in endurance athletes. Contrary to our hypothesis, we found no association between the REDs risk and LDL-C or TC in either female or male athletes in a cross-sectional analysis. Furthermore, we found no overall difference between REDs risk or cholesterol levels between the different phases of the training season. Surprisingly, an increase in the REDs risk from the beginning to the end of the training season was associated with a decrease in LDL-C and TC levels in female athletes.

Our findings are in contrast to the current REDs model, in which elevated LDL-C and TC levels are listed as indicators of problematic LEA.^1 32^ Previous evidence on the topic is mixed, with some studies reporting an association between markers of problematic LEA and elevated LDL-C or TC,^27^,^13 19 33^ while others found no associations.^714^,^18^ This inconsistency may be partly due to differences in the severity and duration of LEA across studies. Extreme and prolonged LEA might disturb cholesterol metabolism in a way that less severe LEA does not. Our study represents the spectrum of severity of LEA in a real-world athlete population and, to our knowledge, is the first to examine the association between cholesterol levels and REDs risk using the IOC REDs CAT 2. However, due to the small number of individuals in the highest risk groups, we had to assess REDs risk dichotomously. Therefore, the medium-to-high REDs risk group includes athletes at mild to severe risk, which may have diluted our results so that effects specific to those at severe risk may have been missed. To account for this heterogeneity, we also looked at the individual REDs indicators and no associations were found with LDL-C or TC.

We found that an increase in REDs risk from the beginning to the end of the training season was associated with a reduction in both LDL-C and TC in female athletes, even after adjustments for change in body fat percentage, FLMR or season of the year. This finding could be due to the acute effects of mild energy restriction on cholesterol levels. Moderate energy restriction lowers LDL-C and TC in the general population independent of obesity,^34 35^ but continued and more severe energy restriction has been associated with elevated LDL-C and TC in military personnel undergoing heavy physical exertion^10^ and in patients with anorexia nervosa.^19^ Physique athletes voluntarily undergoing extreme LEA and weight loss have experienced beneficial but transient changes in HDL-C and very-low density lipoprotein (VLDL), but no changes in LDL-C.^15 36^ In addition, athletes with oligomenorrhoea (irregular menstrual bleeding) have been reported with the most favourable lipid profile compared with athletes with amenorrhoea (no menstrual bleeding) or eumenorrhoea (normal menstrual bleeding), suggesting that mild LEA could have a beneficial effect on cholesterol levels.^2^ These results highlight the complexity of lipid metabolism and the impact of LEA and weight loss on it.

Endurance athletes are susceptible to problematic LEA and REDs due to their high training volume, increased risk of disordered eating and the weight-sensitive nature of their sport.^37 38^ In our study, the prevalence of medium-to-high REDs risk ranged from 72% to 78% in female athletes, depending on the phase of the training season. This is consistent with previous reports, in which 18%–80% of female endurance athletes have been at risk of REDs.^39^,^47^ However, in male athletes, our prevalence rates ranging from 72% to 86% were even higher than the previously reported range from 42% to 70%.^4346^,^49^ The methods used to classify the risk of REDs have varied considerably from study to study.^50^ We used the IOC-recommended REDs CAT 2.

### Clinical implications

The lack of association between the REDs risk and cholesterol levels in the present study suggests that the inclusion of elevated LDL-C and TC in the REDs screening tool may require further evaluation. However, we found that regardless of the REDs risk status, up to 31% of female and 20% of male athletes had clinically elevated LDL-C (≥3 mmol/L). These prevalences correspond to those in the general population of young adults,^51 52^ suggesting that athletes are not protected from dyslipidaemia, but rather have a similar risk to age-matched peers.

### Strengths and limitations

Our study has some important strengths. The participants were national-level to international-level endurance athletes, enabling us to assess REDs risk and cholesterol levels in a highly trained athlete population. We used standard protocols in the measurement of body composition, BMD and blood samples. For assessing the REDs risk, we used the IOC-recommended REDs CAT2 which has been validated by a process of expert validation.^1 24^

We had some limitations in our assessment of REDs risk. First, because data collection began prior to the publication of the REDs CAT2, we did not have all the required information, such as psychiatric assessment for anxiety and depression, or the duration of amenorrhoea. We used the short version of the EDE-Q (EDE-QS) and a common 15-point cut-off for both female and male athletes, as there are no specific cut-offs for males.^22^ The common cut-off might underestimate disordered eating behaviour among men. DXA for BMD assessment was conducted only once during the athletic year and the result was extrapolated to represent both time points within the same annual cycle. However, because changes in BMD usually occur slowly, we believe that significant changes in BMD between the two time points are rare.^53^ Finally, the accuracy of the REDs CAT 2 in screening athletes with REDs requires further study,^54^ and the model remains to be investigated and further developed.^24 54^ Taken together, these limitations may have led to an overestimation or underestimation of the risk of REDs.

We cannot exclude the possibility of unknown confounding factors affecting our findings. Overall, we were powered to detect medium effect sizes. Small but potentially meaningful differences may have gone undetected. We were unable to collect specific training information in T1 and T2, which limits the interpretation of the potential effect of training on cholesterol levels. Future studies could also assess whether there are systematic differences in athletes’ diet within the training season. Genetic factors, potentially influencing cholesterol metabolism, could not be assessed, but no extremely high LDL-C levels (≥4.9 mmol/L) were observed, which would raise concern of familial hypercholesterolaemia.^55^ Although mean cholesterol levels in Finland are typical when compared with other European countries,^56^ further studies are needed to assess the generalisability of our findings to athletes from other sport, cultural and ethnic backgrounds.

## Conclusions

We found no association between REDs risk and elevated levels of LDL-C or TC, suggesting limited value of these lipid parameters in identifying REDs among athletes. The prevalence of elevated LDL-C and TC among athletes was similar to that in the general population. These results suggest that the existing REDs screening tools may need to be re-evaluated and serve as a reminder that even otherwise healthy athletes may be at risk for future cardiovascular disease.

## Supplementary material

10.1136/bmjsem-2025-002644 online supplemental file 1

## Data Availability

Data are available on reasonable request.

## References

[1] Mountjoy M, Ackerman KE, Bailey DM (2023). 2023 International Olympic Committee’s (IOC) consensus statement on Relative Energy Deficiency in Sport (REDs). Br J Sports Med.

[2] Rickenlund A, Eriksson MJ, Schenck-Gustafsson K (2005). Amenorrhea in female athletes is associated with endothelial dysfunction and unfavorable lipid profile. J Clin Endocrinol Metab.

[3] Hooper DR, Kraemer WJ, Saenz C (2017). The presence of symptoms of testosterone deficiency in the exercise-hypogonadal male condition and the role of nutrition. Eur J Appl Physiol.

[4] Meyer MR, Haas E, Barton M (2006). Gender Differences of Cardiovascular Disease. Hypertension.

[5] Palmisano BT, Zhu L, Eckel RH (2018). Sex differences in lipid and lipoprotein metabolism. Mol Metab.

[6] Kaur H, Werstuck GH (2021). The Effect of Testosterone on Cardiovascular Disease and Cardiovascular Risk Factors in Men: A Review of Clinical and Preclinical Data. *CJC Open*.

[7] Silvennoinen JIK, Ihalainen JK, Valtonen M (2024). Association of LEAF-Q and EDE-QS scores with cholesterol levels in Finnish female athletes. BMJ Open Sport Exerc Med.

[8] Friday KE, Drinkwater BL, Bruemmer B (1993). Elevated plasma low-density lipoprotein and high-density lipoprotein cholesterol levels in amenorrheic athletes: effects of endogenous hormone status and nutrient intake. J Clin Endocrinol Metab.

[9] Kaiserauer S, Snyder AC, Sleeper M (1989). Nutritional, physiological, and menstrual status of distance runners. Med Sci Sports Exerc.

[10] Friedl KE, Moore RJ, Hoyt RW (2000). Endocrine markers of semistarvation in healthy lean men in a multistressor environment. J Appl Physiol.

[11] Lamon-Fava S, Fisher EC, Nelson ME (1989). Effect of exercise and menstrual cycle status on plasma lipids, low density lipoprotein particle size, and apolipoproteins. J Clin Endocrinol Metab.

[12] O’Donnell E, De Souza MJ (2004). The cardiovascular effects of chronic hypoestrogenism in amenorrhoeic athletes: a critical review. Sports Med.

[13] Kasper AM, Crighton B, Langan-Evans C (2019). Case Study: Extreme Weight Making Causes Relative Energy Deficiency, Dehydration, and Acute Kidney Injury in a Male Mixed Martial Arts Athlete. Int J Sport Nutr Exerc Metab.

[14] Black K, Slater J, Brown RC (2018). Low Energy Availability, Plasma Lipids, and Hormonal Profiles of Recreational Athletes. J Strength Cond Res.

[15] Jouhki I, Sarin HV, Jauhiainen M (2024). Effects of fat loss and low energy availability on the serum cardiometabolic profile of physique athletes. Scand J Med Sci Sports.

[16] Cardiovascular risk markers in hypothalamic amenorrhoea - Miller - 2000 - Clinical endocrinology. https://onlinelibrary-wiley-com.libproxy.helsinki.fi/doi/full/10.1046/j.1365-2265.2000.01100.x?sid=nlm%3Apubmed.

[17] Thompson DL, Snead DB, Seip RL (1997). Serum Lipid Levels and Steroidal Hormones in Women Runners With Irregular Menses. Can J Appl Physiol.

[18] Kyte KH, Stensrud T, Berg TJ (2022). Vascular Function in Norwegian Female Elite Runners: A Cross-Sectional, Controlled Study. *Sports (Basel*).

[19] Hussain AA, Hübel C, Hindborg M (2019). Increased lipid and lipoprotein concentrations in anorexia nervosa: A systematic review and meta-analysis. Int J Eat Disord.

[20] McKay AKA, Stellingwerff T, Smith ES (2021). Defining Training and Performance Caliber: A Participant Classification Framework. Int J Sports Physiol Perform.

[21] Fairburn CG, Beglin SJ (1994). Assessment of eating disorders: interview or self-report questionnaire?. Int J Eat Disord.

[22] Prnjak K, Mitchison D, Griffiths S (2020). Further development of the 12-item EDE-QS: identifying a cut-off for screening purposes. BMC Psychiatry.

[23] Gideon N, Hawkes N, Mond J (2016). Development and Psychometric Validation of the EDE-QS, a 12 Item Short Form of the Eating Disorder Examination Questionnaire (EDE-Q). PLoS One.

[24] Stellingwerff T, Mountjoy M, McCluskey WT (2023). Review of the scientific rationale, development and validation of the International Olympic Committee Relative Energy Deficiency in Sport Clinical Assessment Tool: V.2 (IOC REDs CAT2)-by a subgroup of the IOC consensus on REDs. Br J Sports Med.

[25] Taipale-Mikkonen RS, Raitanen A, Hackney AC (2021). Influence of Menstrual Cycle or Hormonal Contraceptive Phase on Physiological Variables Monitored During Treadmill Testing. Front Physiol.

[26] Ockene IS, Chiriboga DE, Stanek EJ (2004). Seasonal variation in serum cholesterol levels: treatment implications and possible mechanisms. Arch Intern Med.

[27] Ma X, Yan H, Zhang H (2020). Progress in the seasonal variations of blood lipids: a mini-review. Lipids Health Dis.

[28] Brown VA (2021). An Introduction to Linear Mixed-Effects Modeling in R. Advances in Methods and Practices in Psychological Science.

[29] Bates D, Mächler M, Bolker B (2015). Fitting Linear Mixed-Effects Models Using Lme4. J Stat Softw.

[30] Mansournia MA, Collins GS, Nielsen RO (2021). A CHecklist for statistical Assessment of Medical Papers (the CHAMP statement): explanation and elaboration. Br J Sports Med.

[31] Elm E von, Altman DG, Egger M (2007). Strengthening the reporting of observational studies in epidemiology (STROBE) statement: guidelines for reporting observational studies. BMJ.

[32] (2023). International olympic committee relative energy deficiency in sport clinical assessment tool 2 (IOC REDs CAT2). Br J Sports Med.

[33] Swenne I (2016). Plasma cholesterol is related to menstrual status in adolescent girls with eating disorders and weight loss. Acta Paediatr.

[34] Soare A, Weiss EP, Pozzilli P (2014). Benefits of caloric restriction for cardiometabolic health, including type 2 diabetes mellitus risk. Diabetes Metab Res Rev.

[35] Most J, Gilmore LA, Smith SR (2018). Significant improvement in cardiometabolic health in healthy nonobese individuals during caloric restriction-induced weight loss and weight loss maintenance. Am J Physiol Endocrinol Metab.

[36] Sarin HV, Lee JH, Jauhiainen M (2019). Substantial fat mass loss reduces low-grade inflammation and induces positive alteration in cardiometabolic factors in normal-weight individuals. Sci Rep.

[37] Mancine RP, Gusfa DW, Moshrefi A (2020). Prevalence of disordered eating in athletes categorized by emphasis on leanness and activity type - a systematic review. J Eat Disord.

[38] Palazzo R, Parisi T, Rosa S (2024). Energy Availability and Body Composition in Professional Athletes: Two Sides of the Same Coin. Nutrients.

[39] Melin A, Tornberg ÅB, Skouby S (2015). Energy availability and the female athlete triad in elite endurance athletes. Scand J Med Sci Sports.

[40] Kettunen O, Mikkonen R, Linnamo V (2023). Nutritional intake and anthropometric characteristics are associated with endurance performance and markers of low energy availability in young female cross-country skiers. J Int Soc Sports Nutr.

[41] Muia EN, Wright HH, Onywera VO (2016). Adolescent elite Kenyan runners are at risk for energy deficiency, menstrual dysfunction and disordered eating. J Sports Sci.

[42] Fahrenholtz IL, Melin AK, Wasserfurth P (2022). Risk of Low Energy Availability, Disordered Eating, Exercise Addiction, and Food Intolerances in Female Endurance Athletes. *Front Sports Act Living*.

[43] Jesus F, Castela I, Silva AM (2021). Risk of Low Energy Availability among Female and Male Elite Runners Competing at the 26th European Cross-Country Championships. Nutrients.

[44] Karlsson E, Alricsson M, Melin A (2023). Symptoms of eating disorders and low energy availability in recreational active female runners. BMJ Open Sport Exerc Med.

[45] Dervish RA, Wilson LJ, Curtis C (2023). Investigating the prevalence of low energy availability, disordered eating and eating disorders in competitive and recreational female endurance runners. Eur J Sport Sci.

[46] Viner RT, Harris M, Berning JR (2015). Energy Availability and Dietary Patterns of Adult Male and Female Competitive Cyclists With Lower Than Expected Bone Mineral Density. Int J Sport Nutr Exerc Metab.

[47] McCormack WP, Shoepe TC, LaBrie J (2019). Bone mineral density, energy availability, and dietary restraint in collegiate cross-country runners and non-running controls. Eur J Appl Physiol.

[48] Lane AR, Hackney AC, Smith-Ryan A (2019). Prevalence of Low Energy Availability in Competitively Trained Male Endurance Athletes. Med Bogota Colomb.

[49] Moore EM, Drenowatz C, Stodden DF (2021). Examination of Athlete Triad Symptoms Among Endurance-Trained Male Athletes: A Field Study. Front Nutr.

[50] Gallant TL, Ong LF, Wong L (2025). Low Energy Availability and Relative Energy Deficiency in Sport: A Systematic Review and Meta-analysis. Sports Med.

[51] Trends of elevated low-density lipoprotein cholesterol, awareness, and screening among young adults in the US, 2003-2020 | Cardiology | JAMA cardiology. https://jamanetwork-com.libproxy.helsinki.fi/journals/jamacardiology/fullarticle/2795674.

[52] Zheutlin AR, Luebbe S, Chaitoff A (2024). Low-Density Lipoprotein Cholesterol, Cardiovascular Risk Factors, and Predicted Risk in Young Adults. Clin Cardiol.

[53] Kendler DL, Compston J, Carey JJ (2019). Repeating Measurement of Bone Mineral Density when Monitoring with Dual-energy X-ray Absorptiometry: 2019 ISCD Official Position. J Clin Densitom.

[54] Jeukendrup AE, Areta JL, Van Genechten L (2024). Does Relative Energy Deficiency in Sport (REDs) Syndrome Exist?. *Sports Med*.

[55] Bouhairie VE, Goldberg AC (2015). Familial hypercholesterolemia. Cardiol Clin.

[56] Timmis A, Vardas P, Townsend N (2021). European Society of Cardiology: Cardiovascular Disease Statistics 2021. Eur Heart J.

