# Supplementary material for: Association between the risk of relative energy deficiency in sport and cholesterol levels in Finnish endurance athletes
Source: BMJ Open Sport Exerc Med. 2025 Sep 1;11(3):e002644. doi: 10.1136/bmjsem-2025-002644 (PMC12406806; doi:10.1136/bmjsem-2025-002644)
Supplement: online supplemental file 1 [file bmjsem-11-3-s001.docx]

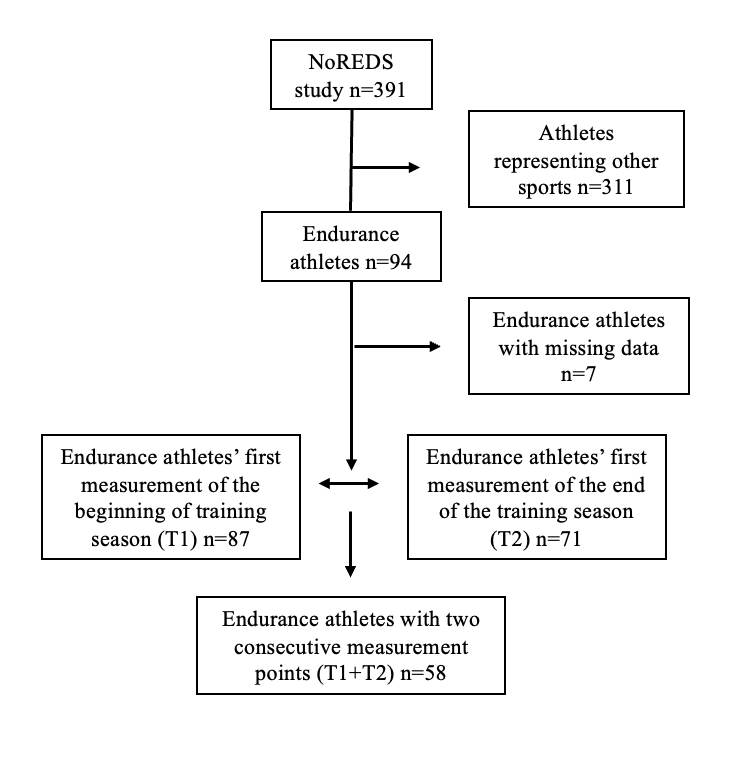


Figure S1. Flowchart of participant selection

Figure S2. REDs risk in female and male athletes who have measurements from the beginning (T1) and end (T2) of their training season

Table S1. Characteristics of study participants who had participated on measurements at the beginning (T1) and end (T2) of the training season

|  |  | female athletes |  |  |  |  | male athletes |  |  |  |
| --- | --- | --- | --- | --- | --- | --- | --- | --- | --- | --- |
|  | **n** | **T1** | **n** | **T2** | **p value** | **n** | **T1** | **n** | **T2** | **p value** |
| age | 27 | 23.8 ±3.7 | 27 | 24.2 ±3.7 | **<0.001** | 31 | 24.9 ±3.5 | 31 | 25.2 ±3.5 | **<0.001** |
| BMI | 27 | 20.6 ±1.7 | 27 | 20.6 ±1.7 | 0.87 | 31 | 22.7 ±1.7 | 31 | 22.8 ±1.5 | 0.24 |
| fat % | 27 | 16.5 [12.0–20.2] | 27 | 16.3 [12.7–20.1] | 0.41 | 31 | 8.3 [6.8–10.8] | 31 | 8.9 [7.1–11.4] | **0.013** |
| FLMR | 27 | 0.20 ±0.08 | 26 | 0.21 ±0.08 | 0.98 | 31 | 0.10 ±0.03 | 31 | 0.11 ±0.04 | **0.019** |
| medium- to-high REDs risk | 27 | 21 (78%) | 27 | 19 (70%) | 0.75 | 31 | 21 (68%) | 31 | 26 (84%) | 0.18 |
| EDE-QS ≥15 | 27 | <5 (<19%) | 27 | 0 | NA | 31 | 0 | 31 | <5 (<16%) | NA |
| BMD ≤-1 | 27 | <5 (<19%) | 27 | <5 (<19%) | NA | 31 | 6 (19%) | 31 | 6 (19%) | NA |
| BSI ≥1 | 27 | 7 (26%) | 27 | <5 (<19%) | 0.25 | 31 | <5 (<16%) | 31 | <5 (<16%) | 1 |
| menstrual disturbances | 22 | 8 (36%) | 19 | <5 (<26%) | 0.16 | *-* | - | *-* | - | - |
| testosterone ≤17nmol/l | - | *-* | - | *-* | - | 31 | 15 (48%) | 31 | 20 (65%) | 0.23 |
| T3 ≤4.25pmol/l | 27 | 17 (63%) | 27 | 17 (63%) | 1 | 31 | 13 (42%) | 31 | 12 (39%) | 1 |
| HDL-C | 27 | 1.6 [1.4–2.0] | 27 | 1.7 [1.6–2.0] | 0.48 | 31 | 1.5 [1.3–1.8] | 31 | 1.5 [1.3–1.8] | 0.53 |
| LDL-C | 27 | 2.4 ±0.7 | 27 | 2.4 ±0.7 | 0.75 | 31 | 2.3 ±0.6 | 31 | 2.5 ±0.6 | 0.062 |
| TC | 27 | 4.4 ±0.9 | 27 | 4.5 ±1.0 | 0.35 | 31 | 4.1 ±0.7 | 31 | 4.3 ±0.8 | 0.092 |
| VO2max | 22 | 59.8 ±5.0 | 23 | 61.4 ±4.6 | **0.002** | 30 | 70.0 ±5.8 | 27 | 70.6 ±6.0 | 0.80 |

# Footnote: Data are mean ±standard deviation, median [interquartile range], or n (%). T1=beginning of the training season, T2=end of the training season, BMI=body mass index (kg/m^2^), FMLR = fat mass to lean mass ratio, REDs=Relative Energy Deficiency in sport, EDE-QS=Eating Disorder Examination Questionnaire Short, BMD=bone mineral density, BSI=bone stress injury, T3 = triiodothyronine, HDL-C=high-density lipoprotein cholesterol (mmol/l), LDL-C=low-density lipoprotein cholesterol (mmol/l), TC=total cholesterol (mmol/l), VO2max=maximal oxygen uptake (ml/kg/min).

Table S2. Association of HDL-C with REDs indicators in female and male athletes in a cross-sectional linear regression model at the beginning (T1) and end (T2) of the training season

| T1 females | n | beta adjusted for age | 95%CI | p value |
| --- | --- | --- | --- | --- |
| menstrual status | 33 | 0.003 | -0.21–0.22 | 0.98 |
| BMD | 44 | -0.10 | -0.22–0.0005 | 0.051 |
| EDE-QS | 44 | -0.00060 | -0.028–0.026 | 0.96 |
| T3 | 44 | -0.034 | -0.12–0.052 | 0.43 |
| T1 males |  |  |  |  |
| testosterone | 43 | -0.026 | -0.057–0.0054 | 0.10 |
| BMD | 43 | 0.13 | -0.002–0.27 | 0.054 |
| EDE-QS | 43 | 0.00012 | -0.037–0.038 | 0.99 |
| T3 | 43 | -0.12 | -0.23– (-0.0084) | **0.035** |
| T2 females |  |  |  |  |
| menstrual status | 28 | -0.06 | -0.47–0.35 | 0.77 |
| BMD | 36 | -0.11 | -0.27–0.032 | 0.12 |
| EDE-QS | 36 | 0.0024 | -0.037–0.042 | 0.90 |
| T3 | 36 | -0.072 | -0.27–0.13 | 0.47 |
| T2 males |  |  |  |  |
| testosterone | 35 | 0.00025 | -0.014–0.014 | 0.97 |
| BMD | 35 | 0.066 | -0.036–0.17 | 0.20 |
| EDE-QS | 35 | 0.0080 | -0.021–0.037 | 0.57 |
| T3 | 35 | -0.075 | -0.14–(-0.0070) | **0.031** |

Footnote: CI=confidence interval, REDs=Relative Energy Deficiency in sport, BMD=bone mineral density, EDE-QS=Eating Disorder Examination Questionnaire, short version, T3=triiodothyronine, menstrual status=eumenorrhea/oligomenorrhea/amenorrhea, use of hormonal contraception excluded

Table S3. Association of LDL-C with REDs indicators in female and male athletes in a cross-sectional linear regression model at the beginning (T1) and end (T2) of the training season

| T1 Females | n | beta adjusted for age | 95%CI | p value |
| --- | --- | --- | --- | --- |
| menstrual status | 33 | 0.17 | -0.18–0.52 | 0.33 |
| BMD | 44 | -0.03 | -0.23–0.17 | 0.76 |
| EDE-QS | 44 | 0.034 | -0.013–0.080 | 0.15 |
| T3 | 44 | 0.14 | -0.006–0.29 | 0.060 |
| T1 males |  |  |  |  |
| testosterone | 43 | 0.025 | -0.023–0.073 | 0.30 |
| BMD | 43 | -0.028 | -0.24–0.19 | 0.80 |
| EDE-QS | 43 | -0.0036 | -0.090–0.018 | 0.18 |
| T3 | 43 | 0.067 | -0.11–0.24 | 0.44 |
| T2 females |  |  |  |  |
| menstrual status | 28 | -0.22 | -1.16–0.71 | 0.62 |
| BMD | 36 | -0.06 | -0.32–0.20 | 0.66 |
| EDE-QS | 36 | -0.0043 | -0.074–0.065 | 0.90 |
| T3 | 36 | 0.066 | -0.30–0.42 | 0.75 |
| T2 males |  |  |  |  |
| testosterone | 35 | -0.0023 | -0.027–0.023 | 0.85 |
| BMD | 35 | 0.16 | -0.021–0.34 | 0.082 |
| EDE-QS | 35 | 0.023 | -0.028–0.075 | 0.36 |
| T3 | 35 | 0.052 | -0.080–0.18 | 0.43 |

Footnote: CI=confidence interval, REDs=Relative Energy Deficiency in sport, BMD=bone mineral density, EDE-QS=Eating Disorder Examination Questionnaire, short version, T3=triiodothyronine, menstrual status=eumenorrhea/oligomenorrhea/amenorrhea, use of hormonal contraception excluded

Table S4 Association of TC with REDs indicators in female and male athletes in a cross-sectional linear regression model in the beginning (T1) and at the end (T2) of the training season

| T1 females | n | beta adjusted for age | 95%CI | p value |
| --- | --- | --- | --- | --- |
| menstrual status | 33 | 0.15 | -0.34–0.64 | 0.55 |
| BMD | 44 | -0.17 | -0.44–0.10 | 0.21 |
| EDE-QS | 44 | 0.03 | -0.038–0.092 | 0.41 |
| T3 | 44 | 0.14 | -0.068–0.35 | 0.18 |
| T1 males |  |  |  |  |
| testosterone | 43 | 0.02 | -0.043–0.08 | 0.55 |
| BMD | 43 | 0.07 | -0.20–0.34 | 0.59 |
| EDE-QS | 43 | -0.05 | -0.12–0.022 | 0.18 |
| T3 | 43 | 0.003 | -0.22–0.22 | 0.98 |
| T2 females |  |  |  |  |
| menstrual status | 28 | -0.26 | -1.45–0.92 | 0.65 |
| BMD | 36 | -0.26 | -0.59–0.085 | 0.14 |
| EDE-QS | 36 | -0.0001 | -0.091–0091 | 0.997 |
| T3 | 36 | 0.05 | -0.42–0.51 | 0.84 |
| T2 males |  |  |  |  |
| testosterone | 35 | -0.004 | -0.038–0.029 | 0.79 |
| BMD | 35 | 0.31 | 0.071–0.53 | 0.012 |
| EDE-QS | 35 | 0.04 | -0.034–0.10 | 0.31 |
| T3 | 35 | -0.02 | -0.20–0.16 | 0.84 |

Footnote: BMD=bone mineral density, EDE-QS=Eating Disorder Examination Questionnaire, short version, T3=triiodothyronine, menstrual status=eumenorrhea/oligomenorrhea/amenorrhea, use of hormonal contraception excluded

Table S5. Multilevel model for the differences in cholesterol values between the beginning (T1) and end of the training season (T2)

|  |  |  |  |  |
| --- | --- | --- | --- | --- |
| females | **n** | **beta adjusted for age** | **95%CI** | **p value** |
| LDL-C | 27 | 0.022 | -0.21–0.25 | 0.85 |
| HDL-C | 27 | 0.076 | -0.078–0.23 | 0.33 |
| TC | 27 | 0.18 | -0.20–0.57 | 0.35 |
| males |  | **beta adjusted for age** | **95%CI** | **p value** |
| LDL-C | 31 | 0.19 | -0.017–0.39 | 0.079 |
| HDL-C | 31 | -0.025 | -0.13–0.077 | 0.63 |
| TC | 31 | 0.23 | -0.054–0.52 | 0.12 |

# Footnote: CI=confidence interval, HDL-C=high-density lipoprotein cholesterol (mmol/l), LDL-C=low-density lipoprotein cholesterol (mmol/l), TC=total cholesterol (mmol/l)

## Table S6. Association of HDL-C, LDL-C, and TC with REDs risk in female athletes not using hormonal contraception in a cross-sectional linear regression model in the beginning (T1) and end (T2) of the training season

|  | | n | beta adjusted for age | 95% CI | p value |
| --- | --- | --- | --- | --- | --- |
|  | T1 | | |  |  |
| HDL-C | | 33 | 0.06 | -0.33–0.44 | 0.77 |
| LDL-C | | 33 | -0.15 | -0.82–0.50 | 0.64 |
| TC | | 33 | -0.29 | -1.19–0.09 | 0.52 |
|  | T2 | | |  |  |
| HDL-C | | 28 | 0.04 | -0.35–0.42 | 0.85 |
| LDL-C | | 28 | -0.01 | -0.82–0.79 | 0.97 |
| TC | | 28 | -0.06 | -1.09–0.98 | 0.91 |

# Footnote: CI=confidence interval, REDs=Relative Energy Deficiency in sport, HDL-C=high-density lipoprotein cholesterol (mmol/l), LDL-C=low-density lipoprotein cholesterol (mmol/l), TC=total cholesterol (mmol/l)

## Table S7. Association of the change in REDs risk from the beginning to the end of the training season with the change in cholesterol values in female athletes not using hormonal contraception

| females |  |  |  |  |  |  |  |
| --- | --- | --- | --- | --- | --- | --- | --- |
| Δ REDs risk | n | beta unadjusted | 95%CI | p value | beta adjusted for age | 95%CI | p value |
| Δ HDL-C | 19 | -0.16 | -0.47–0.14 | 0.28 | -0.24 | -0.55–0.064 | 0.11 |
| Δ LDL-C | 19 | -0.57 | -0.96– (-0.19) | **0.0061** | -0.62 | -1.04– (-0.10) | **0.0054** |
| Δ TC | 19 | -0.79 | -1.51– (-0.08) | **0.032** | -0.95 | -1.69– (-0.21) | **0.015** |

# Footnote: CI=confidence interval, REDs=Relative Energy Deficiency in sport, HDL-C=high-density lipoprotein cholesterol (mmol/l), LDL-C=low-density lipoprotein cholesterol (mmol/l), TC=total cholesterol (mmol/l)

## Table S8. Association of the change in REDs risk from the beginning to the end of the training season with the change in cholesterol values with adjustment for body fat percentage and FLMR

| females |  |  |  |  |  |  |  |
| --- | --- | --- | --- | --- | --- | --- | --- |
| Δ REDs risk | n | beta adjusted for Δ fat% | 95%CI | p value | beta adjusted for Δ FLMR | 95%CI | p value |
| Δ HDL-C | 27 | -0.18 | -0.44–0.079 | 0.17 | -0.18 | -0.44–0.079 | 0.16 |
| Δ LDL-C | 27 | -0.60 | -0.91– (-0.28) | **0.00066** | -0.56 | -0.89– (-0.23) | **0.0020** |
| Δ TC | 27 | -0.79 | -1.36– (-0.21) | **0.0090** | -0.80 | -1.39– (-0.19) | **0.012** |
| males |  |  |  |  |  |  |  |
| Δ REDs risk | n | beta unadjusted | 95%CI | p value | beta adjusted for age | 95%CI | p value |
| Δ HDL-C | 31 | -0.09 | -0.29–0.11 | 0.36 | -0.09 | -0.29–0.11 | 0.37 |
| Δ LDL-C | 31 | 0.20 | -0.21–0.60 | 0.32 | 0.20 | -0.21–0.60 | 0.33 |
| Δ TC | 31 | 0.020 | -0.57–0.61 | 0.95 | 0.018 | -0.57–0.61 | 0.95 |

# Footnote: CI=confidence interval, REDs=Relative Energy Deficiency in sport, HDL-C=high-density lipoprotein cholesterol (mmol/l), LDL-C=low-density lipoprotein cholesterol (mmol/l), TC=total cholesterol (mmol/l), FMLR = fat mass to lean mass ratio

## Table S9. Minimum detectable effects (MDE) in the linear regression model of association of HDL-C, LDL-C, and TC with REDs risk in female and male athletes in the beginning (T1) and end (T2) of the training season

|  | | n | MDE (beta, mmol/l) | MDE (standardized beta) |
| --- | --- | --- | --- | --- |
|  | females T1 | | |  |
| HDL-C | | 44 | 0.40 | 0.44 |
| LDL-C | | 44 | 0.71 | 0.44 |
| TC | | 44 | 0.97 | 0.44 |
|  | females T2 | | |  |
| HDL-C | | 36 | 0.49 | 0.47 |
| LDL-C | | 36 | 0.92 | 0.47 |
| TC | | 36 | 1.18 | 0.47 |
|  | males T1 | | |  |
| HDL-C | | 43 | 0.39 | 0.44 |
| LDL-C | | 43 | 0.58 | 0.44 |
| TC | | 43 | 0.75 | 0.44 |
|  | males T2 | | |  |
| HDL-C | | 35 | 0.45 | 0.48 |
| LDL-C | | 35 | 0.80 | 0.48 |
| TC | | 35 | 1.08 | 0.48 |

# Footnote: REDs=Relative Energy Deficiency in sport, HDL-C=high-density lipoprotein cholesterol (mmol/l), LDL-C=low-density lipoprotein cholesterol (mmol/l), TC=total cholesterol (mmol/l), MDE=minimum detectable effect, beta=unstandardized regression coefficient, standardized beta=standardized regression coefficient

## Table S10. Minimum detectable effects (MDE) in the linear regression model of association of HDL-C, LDL-C, and TC with REDs risk in female and male athletes in the beginning (T1) and end (T2) of the training season

| females |  |  |  |  | |  |  | |  |  |  |  |  |  |
| --- | --- | --- | --- | --- | --- | --- | --- | --- | --- | --- | --- | --- | --- | --- |
| Δ REDs risk | n | MDE (beta, mmol/l) | | | MDE (standardized beta) | | |  |  |  |  |  |  |  |
| Δ HDL-C | 27 | 0.35 | | | 0.53 | | |  |  |  |  |  |  |  |
| Δ LDL-C | 27 | 0.54 | | | 0.53 | | |  |  |  |  |  |  |  |
| Δ TC | 27 | 0.88 | | | 0.53 | | |  |  |  |  |  |  |  |
| males |  |  | | |  | | |  |  |  |  |  |  |  |
| Δ REDs risk | n | MDE (beta, mmol/l) | | | MDE (standardized beta) | | |  |  |  |  |  |  |  |
| Δ HDL-C | 31 | 0.28 | | | 0.51 | | |  |  |  |  |  |  |  |
| Δ LDL-C | 31 | 0.55 | | | 0.51 | | |  |  |  |  |  |  |  |
| Δ TC | 31 | 0.78 | | | 0.51 | | |  |  |  |  |  |  |  |

# Footnote: REDs=Relative Energy Deficiency in sport, HDL-C=high-density lipoprotein cholesterol (mmol/l), LDL-C=low-density lipoprotein cholesterol (mmol/l), TC=total cholesterol (mmol/l), MDE=minimum detectable effect, beta=unstandardized regression coefficient, standardized beta=standardized regression coefficient
